# Working Memory Beats Age: Evidence of the Influence of Working Memory on the Production of Children’s Emotional False Memories

**DOI:** 10.3389/fpsyg.2021.714498

**Published:** 2021-08-18

**Authors:** Chiara Mirandola, Francesca Pazzaglia

**Affiliations:** ^1^Department of Philosophy, Sociology, Education and Applied Psychology, University of Padua, Padua, Italy; ^2^Department of General Psychology, University of Padua, Padua, Italy

**Keywords:** emotion, false memories, working memory, children, confidence

## Abstract

Emotional valence and working memory ability (WM) affect false memories’ production in adults. Whereas a number of studies have investigated the role of emotional valence in children’s tendency to produce spontaneous false memories, individual differences in WM have not been previously included. In the current article, we were interested in investigating whether emotion and WM would interact in influencing the propensity to incur inferential false memories for scripted events. Ninety-eight typically developing children (first-, third-, and eighth- graders) were administered the Emotional false memory paradigm – allowing to study false memories for negative, positive, and neutral events – and a WM task. Results showed that regardless of age, valence influenced false memories’ production, such that positive events protected against incurring distortions. Furthermore, WM interacted with valence, such that children with higher WM abilities produced fewer false memories for negative events. Concerning confidence judgments, only the youngest group of children claimed to be overconfident when committing false memories for negative and neutral events. Results are discussed in terms of the role of individual differences in higher cognitive abilities interacting with the emotional content of to-be-remembered events.

## Introduction

Emotionally laden events typically lead to better memories than mundane events. A deluge of studies proved this memory-enhancing effect in adults (e.g., Kensinger, 2009). Nonetheless, adults are not exempted from memory distortions when retrieving emotional events (e.g., Kensinger and Schacter, 2005; Brainerd et al., 2008b; Gallo et al., 2009; Mirandola et al., 2014b, 2017). Whether children benefit from a similar effect of emotion on memory and what factors may influence the development of false memories for emotional events are still debated. The current study was aimed at investigating the relation between the emotional content of to-be-remembered events and individual differences in higher cognitive processes, such as working memory capabilities, in children’s tendency to falsely remember everyday events.

For neutral material, when false memories stem from elaborating semantically related wordlists [as in the widely used Deese-Roediger-McDermott (DRM) paradigm; Roediger and McDermott, 1995], a developmental reversal emerges in typically developing children: i.e., age-related increases in memory errors. The DRM paradigm consists of the presentation of several wordlists; within each list, words are semantically related to each other (e.g., *sick, nurse*, and *medicine*) and to a critical lure not presented at encoding (e.g., *doctor*). Claiming to remember such critical lures at retrieval represent committing a false memory. Younger children recall or recognize fewer critical lures than older children and adults. This developmental reversal applies also to emotional stimuli (Howe, 2007; Brainerd et al., 2010). Both the associative-monitoring framework (AAT; Howe, 2005, 2008) and the fuzzy-trace theory (FTT; Brainerd et al., 2008a) may explain the developmental reversal. The former predicts that semantically related but non-presented critical lures are activated at encoding more so than do semantically unrelated words, and that this effect is even boosted when the critical lures are emotionally charged; thus, it follows that younger children – whose ability to process semantic relations among words is not fully developed and undergoes important changes due to maturation and education during development – produce false memories to a lesser extent than older peers and adults. The FTT instead predicts a similar developmental increase in false memories since emotionally charged events support gist (i.e., thematic) connections more than neutral events and gist connections are responsible for false memory formation. Thus, it follows that the developing ability to detect gist connections among experienced events leads to increasing production of false memory with increasing age.

A more ecologically valid paradigm, the Emotional False Memory Paradigm (Mirandola et al., 2017) has been recently widely used to test spontaneous false memories for emotionally valenced events in the entire life-span: older adults (Toffalini et al., 2019), adults (Mirandola et al., 2014b, 2017, 2020; Mirandola and Toffalini, 2016), adolescents (Toffalini et al., 2014, 2015) and both typically developing children (Melinder et al., 2017) and children with developmental disorders (Mirandola et al., 2014a; Solomon et al., 2019). This paradigm consists of the presentation of photographs depicting different scripts or episodes at encoding. Each episode may end either in a positive, negative or neutral way. For example, in the dating episode, a boy and a girl are shown while they are getting ready to go out on a date (e.g., getting dressed, combing their hair, text messaging on the cellphone, etc.). The episode may have a positive (the boy and the girl are fiancée, and they kiss each other), negative (the boy is aggressive toward the girl for being late) or neutral (the boy and girl are friends and meet for exchanging a textbook) ending. The causal antecedent – that is the scene depicting what happens in the story right before the episode ending – is not shown during encoding, but it is presented at recognition. Claiming to remember the causal antecedent represents committing an inferential causal error (see Supplementary Figure 1 for the pictorial example of this episode). Another type of error elicited within this paradigm is the gap-filling error, which corresponds to erroneously remembering non-presented but script-consistent pictures (e.g., remembering the girl combing her hair while she was brushing her teeth). The available evidence in typically developing children shows that positive but not negative events protect children – regardless of age – from incurring false memories, especially causal errors (Melinder et al., 2017). The authors suggest that the underdeveloped working memory in children (as young as 6 years in their study) may have played a role. Evidence in adults with the same paradigm (Mirandola et al., 2017) shows that both individual differences in working memory (Exp. 1) and a double task at encoding (Exp. 2) influence false memories for negative events: indeed, individuals with lower WM abilities produce more false memories for negative events, thus discarding the protecting effect of emotion on false memory production. The hypothesized mechanism is that individuals with higher WM are better able to manipulate both useful and irrelevant information, excluding the latter ones when unnecessary for the ongoing task. Given that negative information is more difficult to be inhibited than positive information (Osaka et al., 2013), it follows that people with reduced WM would struggle to manipulate negative events and thus would be more prone to include negative events in their memory, even if not presented (Mirandola et al., 2017). As far as children are concerned, given that younger children have lower WM abilities than older children and adults, one would expect more false memories for negative events in younger children. Melinder et al. (2017) found that regardless of age only positive events protected against false memories and that negative false memories were produced to a similar extent than neutral ones. However, they could only speculate on the possible role of WM given that it was not tested in their sample of children. The current study was aimed at specifically testing the influence of WM abilities on the production of emotional false memories in children. To this end, we administered the Emotional False Memory Paradigm and the Letter-number sequencing WM subtest of the Wechsler Intelligence Scale for Children to three groups of children in order to detect possible developmental changes: 1st graders, 3rd graders, and 8th graders. We could hypothesize that younger children – due to lower WM ability – would produce more false memories for negative events compared to older children. Alternatively, we could hypothesize that regardless of age, WM would interact with emotional valence protecting children from incurring false memories for negative events.

## Methods

### Participants

Ninety-eight typically developing children participated to this study. Specifically, 23 first-grade children (*M*_*age*_ = 82.6, *SD* = 3.4; females = 10), 40 third-grade children (*M*_*age*_ = 105, *SD* = 4.4; females = 20), and 35 eight-grade children (*M*_*age*_ = 156.6, *SD* = 6.6; females = 12). We determined the number of participants on the basis of a power analysis using the G^∗^Power program (Erdfelder et al., 1996), which indicated that a total sample of 87 participants would be needed to detect differences using a repeated measure ANOVA design, with power (1 - β) set at 0.90, alpha at 0.05, and with an estimated η*_*p*_*^2^ = 0.04 (based on previous work with the same false memory paradigm and the same repeated measure design; Mirandola et al., 2017). Inclusion criteria were: Italian as either first language or second language but with advanced knowledge (documented by the teachers) and absence of a diagnosis of learning disorders or other neurodevelopmental disorders (documented by the school records). Due to school restrictions we could not assess participants’ IQ, however, we shall note that the WM task that we employed in this study is included into the Wechsler intelligence scale for children to calculate the total IQ; furthermore, WM ability and IQ are highly correlated (see Giofrè and Cornoldi, 2015). We obtained written informed consent from all children’s parents. The study was approved by the Local Ethical Board of the University of Padua (no. 2232).

### Materials and Procedure

#### Emotional False Memory Paradigm

##### Encoding

A sequence of color photographs depicting nine episodes was employed. The episodes were the following: going grocery shopping, waking up, going to a bike trip, rock climbing, track competition, homecoming after a long trip, dating, birthday party, playing at the slot machine (see Mirandola et al., 2014b, 2017 for pictorial examples of the episodes). For each episode, 14 photographs depicted actions that typically occur during the event (11 were used as target photographs in the encoding phase and three were used as gap-filling distractors in the recognition phase), and two photographs depicted cause-effect scenes (the effect scene was studied whereas the cause scene was presented only during the recognition test). The emotionality of the effect photographs was balanced across episodes, such that the same cause could have three different outcomes: positive, negative, and neutral. Finally, the stimuli also included 10 photographs that were inconsistent with any of the nine episodes, such as children playing on the beach, shown at the beginning and at the end of the presentation in order to avoid primacy and recency effects on the relevant material. Participants saw the nine episodes in sequence without any interruption between them.

Children were tested individually in a quiet room at their school. They were told that they would see several photographs depicting other children and young people doing different daily activities and that they would have to pay close attention and try to understand what the stories represented. The encoding phase consisted of a series of 126 photographs; each photo was presented for 2 s and was followed by a black screen lasting 2 s. The nine episodes were presented – using Microsoft PowerPoint program —in a fixed order, with target-distractor photographs and valence of the episode-ending varying across participants. The encoding phase was followed by a 15-min retention interval. During this interval, children were administered filler tasks.

##### Recognition

Children received a surprise memory test. Stimuli for the recognition phase consisted of a series of 45 target and 45 distractor photographs in a randomized order. For each episode, four targets and four distractors were included (one of the four distractor photographs were the causal antecedent whose outcome had been presented during study). Further, 18 photographs inconsistent with any of the episodes were included (nine targets and nine distractors). The memory test consisted of a self-paced recognition task. For each photograph, children had to utter “yes” or “no” whether they could, respectively, remember having seen the photograph during the encoding phase or not. Furthermore, children had to provide confidence judgments for each response given, using the Confidence Rating Board (CRB), proved effective even with children as young as 5 years.

#### Confidence Rating Board

The Confidence Rating Board (CRB; Ghetti et al., 2002) consists of two photograhps that depict either a child with a confident expression or the same child with a doubtful expression. These photographs are positioned on the opposite sides of a white board. Three dots are drawn between these photographs which represent the three degrees of confidence (very sure, somewhat sure, and not sure at all). Children were instructed to utter how sure they were that they saw/did not see the picture before, using the board as a help; they could only point to the dot near the picture of the child with a confident facial expression when they were very sure (that they saw or that they did not see the photograph), the middle dot, when they were somewhat sure, and the dot near the doubtful facial expression when they were not at all sure. See the photographs used in the CRB in the Supplementary Figure 2.

At the end of the experimental session, participants were also asked to orally describe what the actors of each script were doing and feeling. All children were able to explain in their own words the content of the stories and the feelings of the actors represented.

#### Working Memory Task

After the Emotional False Memory paradigm, children were administered the WM task (Letter-Number Sequencing, Wisc-IV; Wechsler, 2004). The WM task consists of the presentation of a sequence of digits and letters in “scrambled” order; the child is required to immediately repeat first the digits from the smallest to the biggest, and second the letters in alphabetical order. Sequences of alphanumerical elements increase from two to ten strings. The task is self-paced, such that after the erroneous repetition of two strings within the same sequence, it is interrupted and the corresponding WM span is calculated.

## Results

### Working Memory

A univariate ANOVA revealed a main effect of Grade (1st vs. 3rd vs. 8th) on the WM span, *F*(2,95) = 25.53, *p* < 0.001, η*_*p*_^2^* = 0.35, showing a clear developmental improvement: 8th graders had a significantly higher WM span (*M* = 18.83, *SD* = 3.28) than 3rd graders (*M* = 16.15, *SD* = 2.25), who in turn had a significantly higher WM span than 1st graders (*M* = 13.52, *SD* = 2.82).

### False Memories

**FIGURE 1:**
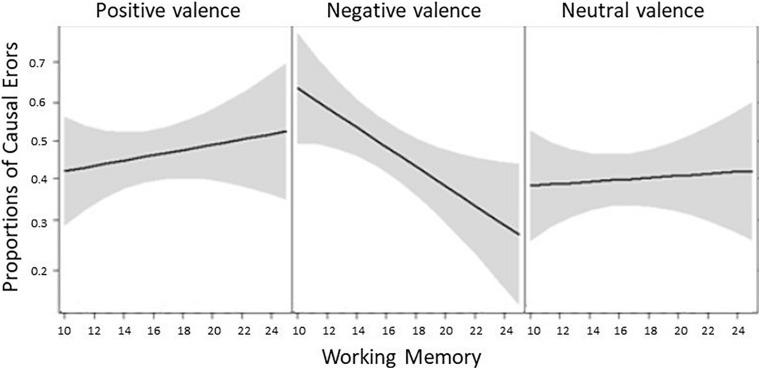
Mean proportions of false memories (i.e., causal errors) as a function of emotional valence (i.e., positive, negative, and neutral) and WM abilities. Shaded gray areas represent 95% CIs.

Within the Emotional False Memory paradigm two types of false memories were calculated, namely causal errors and gap-filling errors. Mean proportions of “yes” responses to causal distractor images represent causal errors, whereas mean proportions of “yes” responses to script-consistent distractor images represent gap-filling errors. Preliminary analysis with gender as the between- participant factor and proportions of either causal errors (*p* = 0.44) and gap-filling errors (*p* = 0.25) as the dependent measures, revealed no main effect. Thus, gender was not included as a covariate in the following analyses. A linear model (ANCOVA) was computed with Grade (1st vs. 3rd vs. 8th) as the between-participants factor, Valence (positive vs. negative vs. neutral) as the within-participants factor and WM score (continuous variable treated as a covariate) over the proportion of causal errors as the dependent measure. A main effect of Valence was found, *F*(2,188) = 3.95, *p* = 0.02, η*_*p*_^2^* = 0.04, such that regardless of age, causal errors for positive events (*M* = 0.39, *SD* = 0.28) were produced to a lesser extent than both negative (*M* = 0.46, *SD* = 0.32) and neutral (*M* = 0.47, *SD* = 0.28) events. Furthermore, a significant interaction between Valence and WM emerged, *F*(2,188) = 3.70, *p* = 0.03, η*_*p*_^2^* = 0.04. As a *post hoc* analysis for this interaction we calculated single correlations between WM and each of the three levels of valenced causal errors. Only the correlation between WM and negative causal errors was significant (*r* = −0.25, *p* = 0.01; positive causal errors: *r* = −0.07, *p* = 0.48; neutral causal errors: *r* = −0.09, *p* = 0.33) (see Figure 1). The main effect of Grade was not found, *F*(2,94) = 0.77, *p* = 0.46, η*_*p*_^2^* = 0.01.

We then computed a similar analysis with grade, valence, and WM as the predictors and gap-filling errors as the dependent measure. No main effect of Valence [*F*(2,188) = 0.05, *p* = 0.95, η*_*p*_^2^* = 0.001] nor interactive effect with Grade [*F*(4,188) = 0.68, *p* = 0.60, η*_*p*_^2^* = 0.01] and WM [*F*(2,188) = 0.08, *p* = 0.92, η*_*p*_^2^* = 0.001] emerged. We found a main effect of Grade, *F*(2,94) = 3.14, *p* = 0.048, η*_*p*_^2^* = 0.06, with the tendency of older children to produce fewer gap-filling errors than 8-year-old children (8th graders: *M* = 0.20, *SD* = 0.13; 3rd graders: *M* = 0.28, *SD* = 0.16; 1st graders: *M* = 0.23, *SD* = 0.12); however, Bonferroni-corrected *post hoc* comparisons did not reach statistical significance (*p* = 0.073).

### Confidence Judgments Associated With False Memories

**FIGURE 2:**
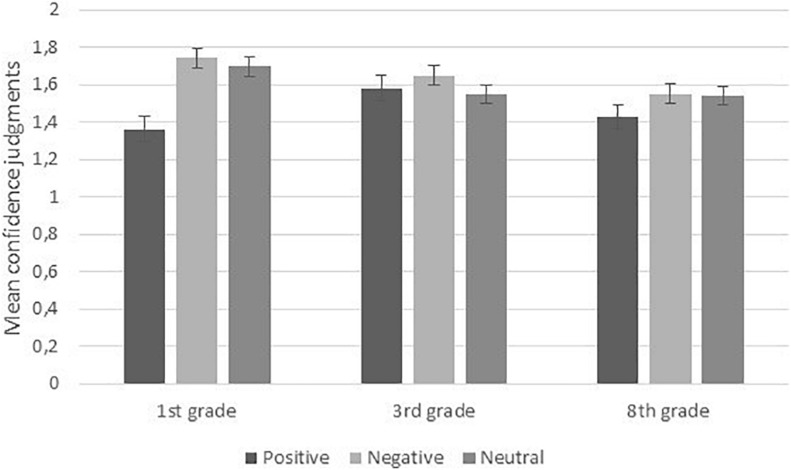
Mean confidence judgments associated with causal errors as a function of emotional valence and grade. Bars represent standard errors.

When we computed the analysis on the mean confidence judgments associated with causal errors, the main effect of Valence [*F*(2,186) = 0.65, *p* = 0.52, η*_*p*_^2^* = 0.007], the main effect of Grade [*F*(2,93) = 0.59, *p* = 0.55, η*_*p*_^2^* = 0.01] nor the interactive effect between Valence and WM [*F*(2,186) = 1.43, *p* = 0.24, η*_*p*_^2^* = 0.01] did not emerge. Interestingly, the interaction between Valence and Grade was significant, *F*(4,186) = 2.39, *p* = 0.05, η*_*p*_^2^* = 0.05; Bonferroni-corrected *post hoc* comparisons showed that only within first Grade did children claim to be overconfident when committing causal errors for negative (*p* = 0.001) and neutral events (*p* = 0.01), compared to positive ones (see Figure 2). A similar analysis conducted on the mean confidence judgments associated with gap-filling errors did not show any main nor interactive effect (all *p*s > 0.34).

### Accuracy

An analysis of variance with Grade as the between-participant factor, WM as the covariate and the “yes” responses to target photographs (i.e., hit rate) as the dependent measure did not reveal the main effect of Grade [*F*(2,94) = 0.63, *p* = 0.53, η*_*p*_^2^* = 0.01] nor WM [*F*(1,94) = 1.63, *p* = 0.21, η*_*p*_^2^* = 0.01]. A similar analysis conducted over the mean confidence judgments for the hit rate did not show any significant effect (all *F*s < 1).

## Discussion

With the current article, we wanted to deepen the understanding of the relation between emotion and WM abilities on the creation of false memories during development. Two hypotheses were put forward: (1) younger children could produce more false memories for negative events, given their reduced WM, compared to older children; and (2) regardless of age, WM could play a stronger influence, with decreasing production of false memories for negative events with increasing WM abilities in all children. We found that individual differences in WM indeed interact with emotion and influence false memory formation to a different extent. Specifically, regardless of age, all children with higher WM abilities produced fewer causal errors for negative events, sustaining the second hypothesis. This finding replicates what previously found in the adult population (Mirandola et al., 2017). Thus, even during development, individual differences in WM are relevant in determining a different tendency to distort memories. Working memory allows to attend to a selection of stimuli while controlling for interfering/irrelevant information (e.g., De Beni et al., 1998; Engle, 2002). We argue that within the current paradigm the negative non-presented events are inferred at encoding, thus becoming interfering information into the newly formed memory trace. People with a lower ability at manipulating the inferred information and later excluding it from memory, incorporate negative false events into their memory and are no longer able to distinguish them from true events. It follows, that people with a lower WM produce a higher amount of false memories for negative events. The current article suggests that this reasoning may be applied to a developmental sample as well. We may hypothesize that the central executive component of Baddeley’s WM model (for a more recent review see Baddeley, 2012) plays an important role; indeed, the central executive has the function to control the processes at hand in ongoing complex tasks (such as the Letter-number sequencing used in the current study which requires children to simultaneously process letters in their alphabetical order and numbers from the smallest to the biggest). Individuals with higher ability in terms of multi-task processing (possible through the central executive) should be better able to distinguish target/experienced events from inferred but not experienced ones while performing an episodic memory task (see also Gómez et al., 2018 for a thorough review of the neurodevelopment of working memory). Furthermore, these findings suggest the need for working memory training in typically developing children with lower WM abilities or in children with disabilities who have hindered WM capacity, in order to investigate the potential benefit on episodic memory, in terms of both accuracy and false recognitions (see Capodieci et al., 2019 for WM training in typically developing children and children with ADHD; Cornoldi et al., 2015 for WM and metacognitive training in typically developing children; and Fraser and Cockcroft, 2020 for WM intervention in special populations of adolescents).

The current work also replicated previous findings, that positive emotional valence *per se* – but not negative valence – protects children from incurring false memories (Melinder et al., 2017). In particular, the effect of emotional valence pertained to causal errors and not gap-filling errors – as found in previous research with the same paradigm (Mirandola et al., 2014b, 2017; Melinder et al., 2017). This is easily explained by the fact that causal photographs are directly linked to the emotional consequence of the episodes, whereas gap-filling errors are not. We did not find developmental differences in causal errors, again replicating available evidence with the same paradigm in typically developing children (Melinder et al., 2017), nor in the accurate recognition of target photographs (i.e., hit rate). While it was possible to hypothesize increasing accuracy relative to correct recognitions of target photographs we must specify that the current Emotional false memory paradigm is specifically designed to elicit false memories and it does not usually lead to developmental differences in accuracy measures nor other effects of the variables of interest on overall memory accuracy (e.g., Mirandola et al., 2014a, 2017).

Another interesting finding concerns the qualitative nature of memory, that is confidence judgments relative to false memories. Younger children did claim to be very confident after falsely recognizing both negative and neutral events – but not positive ones – suggesting again that emotion does not influence only the quantitative aspects of memory, but also the qualitative ones. Previous evidence with the same Emotional false memory paradigm in atypical development (Mirandola et al., 2014a)–but that investigated subjective remembering through the Remember-know paradigm – showed that children with non-verbal learning disabilities claimed to subjectively remember causal errors to a higher extent than typically developing children, regardless of emotional content. These findings suggest that children with disabilities not only produce more false memories, but when that happens, they also associate a higher subjective feeling of vividness to non-experienced events. In the current study, only younger children associated a higher subjective confidence to their negative and neutral false memories compared to the positive ones, suggesting that non-experienced but inferred negative events may be subjectively more compelling than positive ones in younger children. This is conceptually similar to the higher subjective judgments of recollection-based false memories in children with developmental disabilities (Mirandola et al., 2014a).

Taken together, these results show that when studying emotional memory for everyday events, children are protected against distortions only when facing positive events, but not when facing negative events, which are produced to a similar extent than the neutral non-emotional ones. From an applied forensic perspective, it is worth noting the relevance of this evidence. We may expect that children incorporate more false negative events (such as maltreatment episodes) into their memory traces compared to positive ones. However, working memory abilities play an important protecting role in this mechanism, such that even during development, children who have higher WM abilities may benefit from this, reducing their tendency to incur memory distortions for negative events.

## Data Availability Statement

The datasets presented in this study can be found in online repositories. The names of the repository/repositories and accession number(s) can be found below: Data are available on Figshare at doi: 10.6084/m9.figshare.14672622.

## Ethics Statement

The studies involving human participants were reviewed and approved by Ethical Committee for the Psychological Research of the University of Padua. Written informed consent to participate in this study was provided by the participants’ legal guardian/next of kin.

## Author Contributions

CM and FP contributed to the conception and design of the study and read and approved the submitted version. CM organized the dataset, performed the statistical analysis, and wrote the first draft of the manuscript. FP contributed to manuscript revision. Both authors contributed to the article and approved the submitted version.

## Conflict of Interest

The authors declare that the research was conducted in the absence of any commercial or financial relationships that could be construed as a potential conflict of interest.

## Publisher’s Note

All claims expressed in this article are solely those of the authors and do not necessarily represent those of their affiliated organizations, or those of the publisher, the editors and the reviewers. Any product that may be evaluated in this article, or claim that may be made by its manufacturer, is not guaranteed or endorsed by the publisher.
