# Supplementary figures and images for: Working Memory Beats Age: Evidence of the Influence of Working Memory on the Production of Children’s Emotional False Memories
Source: Front Psychol. 2021 Aug 18;12:714498. doi: 10.3389/fpsyg.2021.714498 (PMC8416354; doi:10.3389/fpsyg.2021.714498)

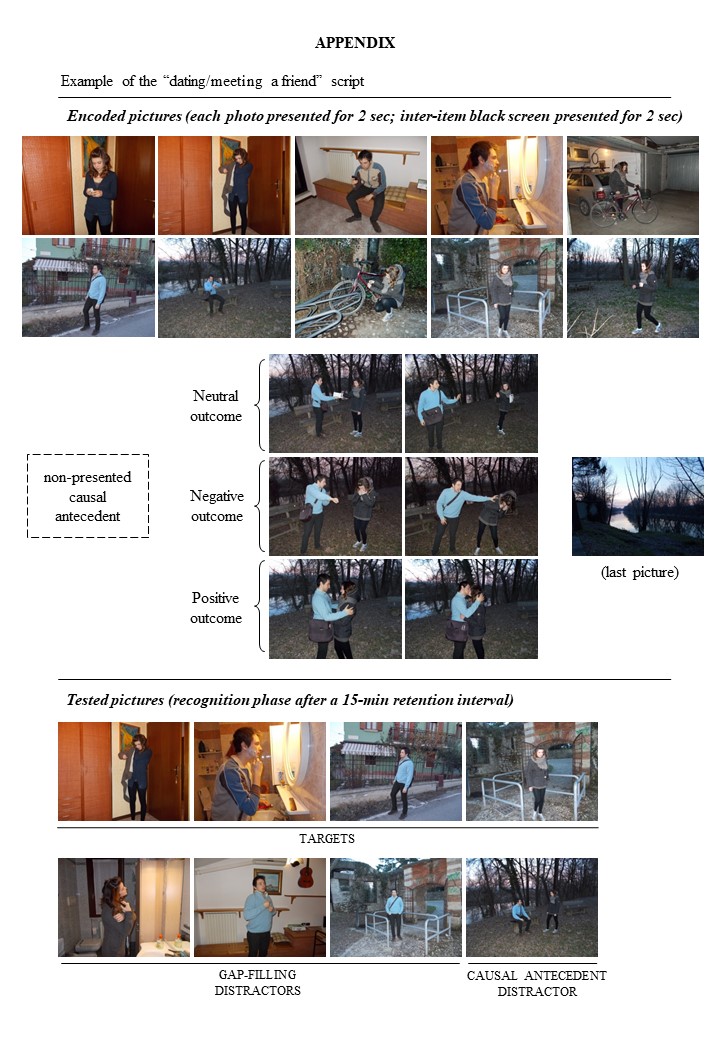

Supplement: Supplementary file 1 [file Image_1.JPEG]

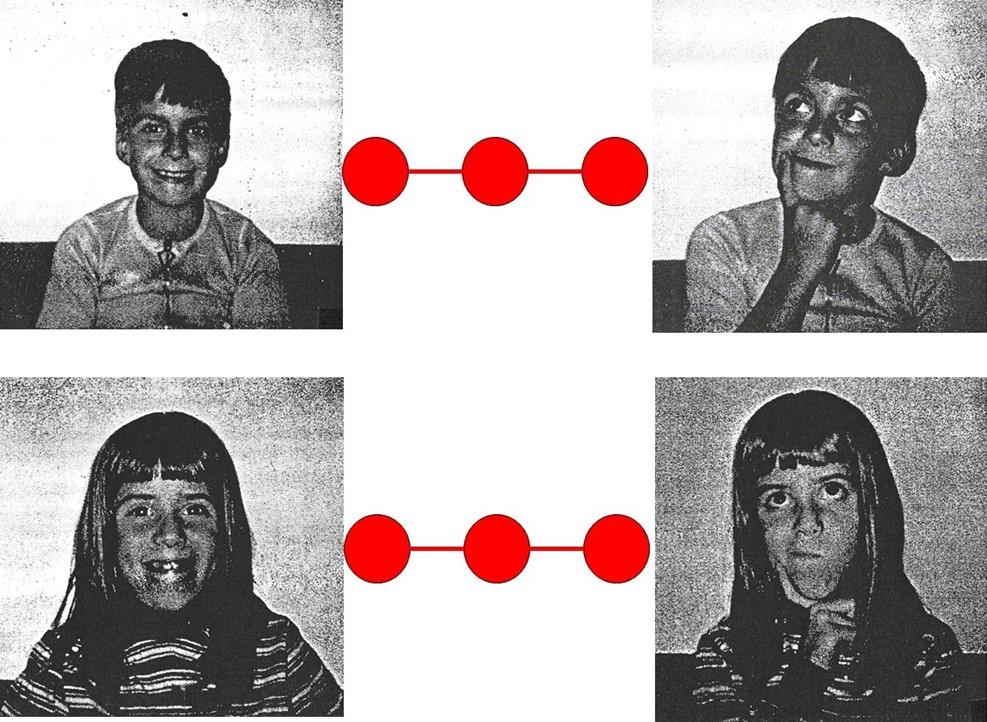

Supplement: Supplementary file 2 [file Image_2.JPEG]
